# Hydraulic niche utilization by larvae of the three Drusinae clades (Insecta: Trichoptera)

**DOI:** 10.2478/s11756-020-00648-y

**Published:** 2020-11-11

**Authors:** Johann Waringer, Simon Vitecek, Jan Martini, Carina Zittra, Stephan Handschuh, Ariane Vieira, Hendrik C. Kuhlmann

**Affiliations:** 1 Department of Functional and Evolutionary Ecology, Division Limnology, University of Vienna, Althanstrasse 14, A-1090 Vienna, Austria; 2 WasserCluster Lunz, Dr. Carl Kupelwieser Promenade 5, A-3293 Lunz am See, Austria; 3 VetCore Facility for Research, Imaging Unit, University of Veterinary Medicine, Veterinärplatz 1, A-1210 Vienna, Austria; 4 Institute of Fluid Mechanics and Heat Transfer, TU Wien, Tower BA/E322, Getreidemarkt 9, A-1060 Vienna, Austria

**Keywords:** Trichoptera larvae, *Drusus spp*., Hydraulic niche, Flow velocity, Drag, Reynolds number, Froude number

## Abstract

Hydraulic niche descriptors of final instar larvae of nine *Drusus* species (Trichoptera) were studied in small, spring-fed, first-order headwaters located in the Mühlviertel (Upper Austria), Koralpe (Carinthia, Austria), and in the Austrian and Italian Alps. The species investigated covered all three clades of Drusinae: the shredder clade (*Drusus franzi*, *D. alpinus*), the grazer clade (*D. biguttatus*, *D. chauvinianus*, *D. dudor*, *D. monticola*), and the filtering carnivore clade (*D. chrysotus*, *D. katagelastos*, *D. muelleri*). Flow velocity was measured at front center of 68 larvae, head upstream, on the top of mineral substrate particles at water depths of 10–30 mm, using a tripod-stabilized Micro propeller meter (propeller diameter = 10 mm). Each data series consisted of a sampled measurement lasting 30 s (measuring interval = 1 s). In total, 2040 single velocity measurements were taken. Instantaneous flow velocities and drag at the sites of the 68 larvae varied from 0 to 0.93 m s^−1^ and 0 to 8346 *10^−6^ N, respectively. Flow velocities and drag between the three clades were highly significantly different (*p* < 0.001); mean velocity (+ 95% confidence limits) for the three clades were 0.09 + 0.00 m s^−1^ for the shredder, 0.25 + 0.00 m s^−1^ for the grazer, and 0.31 + 0.01 m s^−1^ for the filtering carnivore clade; the corresponding data for drag were (85 + 18)*10^−6^ N, (422 + 61)*10^−6^ N and (1125 + 83)*10^−6^ N, respectively. Adhesive friction ranged from (41.07 + 53.03)*10^−6^ N in *D. franzi* to (255.24 + 216.87)*10^−6^ N in *D. chrysotus*. Except in *D. franzi* and *D. dudor* adhesive friction was always well below drag force, indicating that submerged weight alone was not sufficient to stabilize the larvae in their hydraulic environment. Reynolds numbers varied between 0 in *D. franzi* and *D. alpinus*, and 12,634 in *D. katagelastos*, with 7% of the total in the laminar (*R* < 500), 30% in the transitional (*R* = 500–2000), and 61% in the fully turbulent stage (*R* > 2000). Froude numbers (*Fr*) varied from 0 to 2.97. The two *Drusus* species of the shredder clade and three out of four species of the grazer clade were exposed to subcritical *Fr* < 1, one species of the grazer clade and two out of three species of the filtering clade to supercritical Froude numbers >1.

## Introduction

Hydraulic stress exerted on lotic organisms by the unidirectional flow in streams and rivers can be resisted (1) passively by their submerged weight modified by a roughness coefficient, and (2) actively by their efforts to maintain substrate contact (concept of drift resistance; Waringer 1989a). The most important components of hydraulic stress for benthic organisms are lift and drag forces (Statzner and Holm 1982; Statzner et al. 1988; Waringer 1989a, b, 1993; Ditsche-Kuru et al. 2010). The former depends on differences in hydrodynamic pressure ventrally and dorsally of the submerged specimen, modified by body shape (Weissenberger et al. 1991). The ecological implication of drag depends on the organismic Reynolds number *R* where low numbers indicate a high proportion of viscous drag acting at the exposed body surface whereas the proportion of dynamic pressure drag acting at the projected body cross section directed towards flow increases with increasing *R*.

Effectively resisting hydraulic stress and avoiding a risky drift entry is of particular importance for aquatic biota inhabiting steep, shallow stream channels where flow is turbulent and flow velocity is high, the typical habitats of the caddisfly subfamily Drusinae. The subfamily can be divided into three major clades, substantiated by larval morphology and feeding ecologies: omnivorous shredders are fitted with teeth along mandible edges (Graf et al. 2009). Spoon-shaped, teethless mandibles are present in the second group where larvae feed on biofilms and epilithic algae; in some of them, the hydraulic field around their heads is modified by spinule areas (length up to 0.03 mm) posterior to each eye or by additional spines (length 0.4 mm or more) on head and/or pronotum. Finally, larvae of filtering carnivores combine toothed mandibles with filtering spines on legs and the first abdominal sternum; in addition, head capsules are fitted with concavities, in some species combined with a lanuginose hair cover, unknown from other caddisfly larvae (Bohle 1983; Pauls et al. 2008; Vitecek et al. 2015; Waringer et al. 2010, 2015). Such structures have been hypothesized to increase hydraulic stress, as shown for abiotic topographies (Martinuzzi and Tropea 1993; Hwang and Yang 2004; Sedighi and Farhadi 2006).

The present study offers new data on frequently-used hydraulic stress parameters, based on series of in-situ velocity measurements in the immediate harsh, high-alpine hydraulic environment for larval Drusinae, covering the full spectrum of clades outlined above. In detail, (1) we aimed to explore the hydraulic niches of Drusinae larvae belonging to each of the three phylogenetic clades, and (2) to relate this database with models of longitudinal zonation patterns in streams, based on the differences in functional feeding guilds within the three Drusinae clades.

## Materials and methods

### Field measurements

Data were collected in the field from 7 June to 24 July 2019 in small, spring-fed, first order tributaries at elevations of 499 to 2222 m a.s.l. located in the Mühlviertel (Upper Austria), Koralpe (Carinthia, Austria), and in the Austrian and Italian Alps (Table 1). The steep, unpolluted, shallow and summer-cold mountain brooks were 50 to 100 cm wide, with adjoining grassland. Data were taken at the locations of final instar larvae of nine Drusinae species representing the three clades of the subfamily as defined by Pauls et al. (2008), Graf et al. (2009) and Vitecek et al. (2015). In final instar larvae of the nine Drusinae species (maximum head width 1.17 to 1.80 mm; Waringer and Graf 2011), the mineral cases are 9.96–15.05 mm long (Table 2), slightly curved and conical (mean anterior case diameter = 2.76–4.24 mm), creating mean projected frontal areas of 6.01–14.21 mm^2^. Typically, larvae are aligned with flow, with heads directed upstream and their dorsal case outlines situated 5 to 7 mm above the sediment surface. Larvae were mostly encountered at the top of flattened coarse sediment particles (grain size >20 cm) and at water depths ranging from 10 to 30 mm.

**Table 1:** Sampling sites of the nine Drusinae species investigated; showing the number of larvae where velocity measurements were conducted (N), the valency point distribution, and the dates, locations and shear stress where data were collected

Clade / Species	N	Valency points (Hypo-) Krenal /Rhithral	Date	Location (latitude, longitude, elevation; shear stress (N m^−2^))
Shredder clade				
*Drusus alpinus* Meyer-Dür, 1875	7	10 / 0	24 July 2019	Piemonte, Lago del Gias del Prete, Italy (45°31’ N, 07°38′ E, 2222 m; 83 N m^−2^)
*Drusus franzi* Schmid, 1955	9	10 / 0	7 June 2019	Schwarze Sulm, Weinebene, Austria (46°50’ N, 15°01′ E, 1580 m; 14 N m^−2^)
Grazer clade				
*Drusus biguttatus* (Pictet, 1834)	9	0 / 10	7 June 2019	Schwarze Sulm, Weinebene, Austria (46°50’ N, 15°01′ E, 1580 m; 10 N m^−2^)
*Drusus chauvinianus* (Stein, 1874)	3	0 / 10	9 June 2019	Haslach an der Mühl, Austria (48°34′, 14°02′ E, 499 m; 1 N m^−2^)
*Drusus dudor* Oláh, 2017	3	10 / 0	13 June 2019	Valchiusella, Alpe Strup, Italy (45°31’ N, 7°39′E, 1560 m; 38 N m^−2^)
*Drusus monticola* McLachlan, 1876	6	7 / 3	8 June 2019	Schwarze Sulm, Weinebene, Austria (46°50’ N, 15°01′ E, 1580 m; 14 N m^−2^)
Filtering clade				
*Drusus chrysotus* (Rambur, 1842)	5	8 / 2	8 June 2019	Schwarze Sulm, Weinebene, Austria (46°50’ N, 15°01′ E, 1580 m: 14 N m^−2^)
*Drusus katagelastos* Vitecek, 2020	19	7 / 3	11 June 2019	Piemonte, Fondo, Italy (45°30’ N, 07°42′ E, 1584 m; 39 N m^−2^)
*Drusus mülleri* McLachlan, 1868	7	10 / 0	24 July 2019	Piemonte, Lago del Gias del Prete, Italy (45°31’ N, 07°38′ E, 2222 m; 83 N m^−2^)

Valency points for longitudinal zonation patterns within the stream continuum (Krenal/Hypokrenal, Rhithral) from Graf et al. (2008); valency points for *D. dudor* extracted from Vitecek et al. (2020)

**Table 2:** Biometric parameters of nine Drusinae species; showing case dimensions (mm), volumes including cases (mm^3^), fresh and submerged weights (mg), and calculated adhesive frictions (10^−6^ N)

Clade / Species	Anterior case diameter (mm)	Case length (mm)	Projected frontal surface area (mm^2^)	Fresh weight with case (mg)	Volume with case (mm^3^)	Submerged weight (mg)	Adhesive friction (*10^−6^ N)
Shredder clade							
*Drusus alpinus* Meyer-Dür, 1875	2.76 + 0.21	9.96 + 1.09	6.01 + 0.89	44.00 + 12.42	38.00 + 16.19	6.07 + 7.83	41.07 + 53.03
*Drusus franzi* Schmid, 1955	3.38 + 0.42	12.85 + 2.89	9.00 + 2.16	78.50 + 19.34	60.00 + 25.98	10.09 + 0.05	68.29 + 0.31
Grazer clade							
*Drusus biguttatus* (Pictet, 1834)	3.16 + 0.24	10.67 + 1.28	7.92 + 1.28	47.64 + 6.33	42.73 + 11.67	8.46 + 5.43	57.27 + 32.24
*Drusus chauvinianus* (Stein, 1874)	2.86 + 0.17	10.58 + 2.34	6.44 + 0.75	54.40 + 23.65	40.00 + 17.56	14.47 + 13.77	97.95 + 93.16
*Drusus dudor* Oláh, 2017	3.13 + 0.26	10.52 + 0.92	7.76 + 1.32	52.00 + 17.05	45.00 + 19.63	10.47 + 5.64	70.90 + 38.23
*Drusus monticola* McLachlan, 1876	3.50 + 0.20	12.68 + 0.78	9.65 + 1.08	88.33 + 26.33	66.67 + 26.67	21.79 + 9.63	147.46 + 65.15
Filtering clade							
*Drusus chrysotus* (Rambur, 1842)	4.24 + 0.43	13.74 + 1.91	14.21 + 2.76	138.40 + 50.57	102.00 + 28.31	37.71 + 32.05	255.24 + 216.87
*Drusus katagelastos* Vitecek, 2020	3.50 + 0.25	13.17 + 1.60	9.64 + 1.37	86.00 + 2.48	66.67 + 14.34	19.45 + 12.23	131.66 + 82.77
*Drusus muelleri* McLachlan, 1868	4.20 + 0.26	15.05 + 0.20	13.89 + 1.72	110.00 + 7.97	85.00 + 9.19	25.15 + 6.62	170.24 + 44.78

For flow velocity measurements (to the nearest 0.01 m s^−1^), a Schiltknecht MiniWater 20 Micro propeller meter (propeller diameter = 10 mm; time resolution = 1 measurement per s) was firmly attached to a custom-built tripod support, ensuring that the measuring head nearly touched the bottom directly at front center of the larvae. This setup yielded good approximations to the vertically averaged velocity the larva is exposed to (Kuhlmann 2014). Each measurement consisted of a logger-recorded time series with sampling rate of 1 per second, lasting 30 s. In total, 68 time series consisting of 2040 single velocity measurements were used for the present study.

### Biometric parameters

Case lengths and anterior case diameters of a total of 47 final instar larvae of the nine *Drusus* species were measured to the nearest 0.01 mm, using an ocular micrometer attached to a dissecting microscope. Instar determination was based on data by Waringer and Graf (2011) and Vitecek et al. (2020). For the calculation of the projected frontal surface area the anterior case diameter was used. Before weighing larvae in their cases on a laboratory balance (= total fresh weight; to the nearest mg), excess preservative medium was removed with soft tissue. Finally, total volume data for larvae in their cases (to the nearest 0.01 ml) were obtained using a burette filled with water and by taking the difference between volume readings with and without larvae. The biometric data base is included in Table 2.

Adhesive friction *F*_*a*_ is given by the following equation

1 $$ {F}_a=f\  Vg\ \left({\rho}_l-\rho \right) $$

where V is the volume (larva plus case; m^3^), g is the acceleration due to gravity (9.81 m s^−2^), *ρ*_*l*_ is the density of the larva + case (kg m^−3^), *ρ* is the density of water, and *f* is the friction factor which is 0.69 for mineral Trichoptera cases on a mineral substrate (Waringer, 1989a, 1993).

### Hydraulic stress parameters

Hydraulic drag *F*_*d*_ (N) is given by

2 $$ {F}_d={C}_d\ {A\rho u}^2/ 2 $$

where *A* is the projected frontal area, based on mean anterior case diameter (m^2^), *ρ* (kg m^−3^) is the density of water (temperature dependent; for our study streams: *ρ*_*10.5°C*_ = 999.681 kg m^−3^), *u* is the mean flow velocity (m s^−1^) over area A, and *C*_*d*_ is the drag coefficient (for final instar *Drusus* larvae aligned with flow, *C*_*d*_ = 2; Waringer, 1989a, b, 1993).

The dimensionless Froude number *Fr* quantifies the relationship between mean kinetic energy (*u*, as mean velocity) and the potential energy gain across the water depth *d*:

3 $$ Fr=u/{(dg)}^{0.5} $$

with *(dg)*^0.5^ representing the phase velocity of gravity waves in shallow water (subcritical flow: *Fr* < 1.0, critical flow: *Fr* = 1.0, supercritical flow: *Fr* > 1.0; Statzner et al. 1988). Finally, the dimensionless organismic Reynolds number *R* is defined by:

4 $$ R= ul/\nu $$

where *ν* is the temperature-dependent viscosity of water (*ν*_*10.5°C*_ *=* 1.31 * 10^−6^ m^2^ s^−1^), and *l* is larval length (longitudinal axis aligned with flow).

## Results

**Fig. 1:**
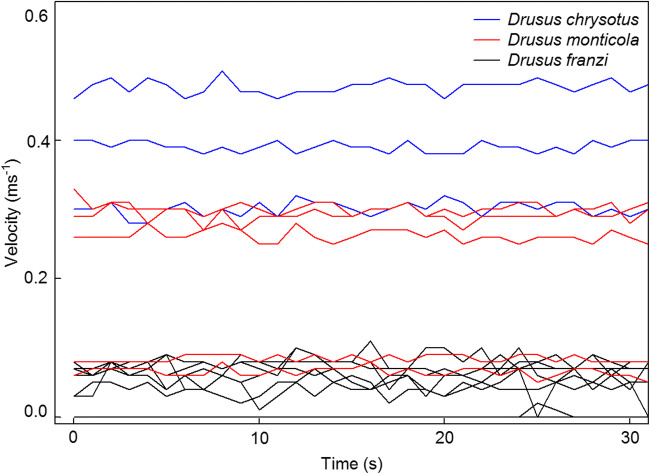
Examples of flow velocity measurements (intervals of 1 s over a period of 30 s) at front center of Drusinae larvae: *Drusus franzi* (shredder clade), *D. chrysotus* (filtering clade), and *D. monticola* (grazer clade)

**Fig. 2:**
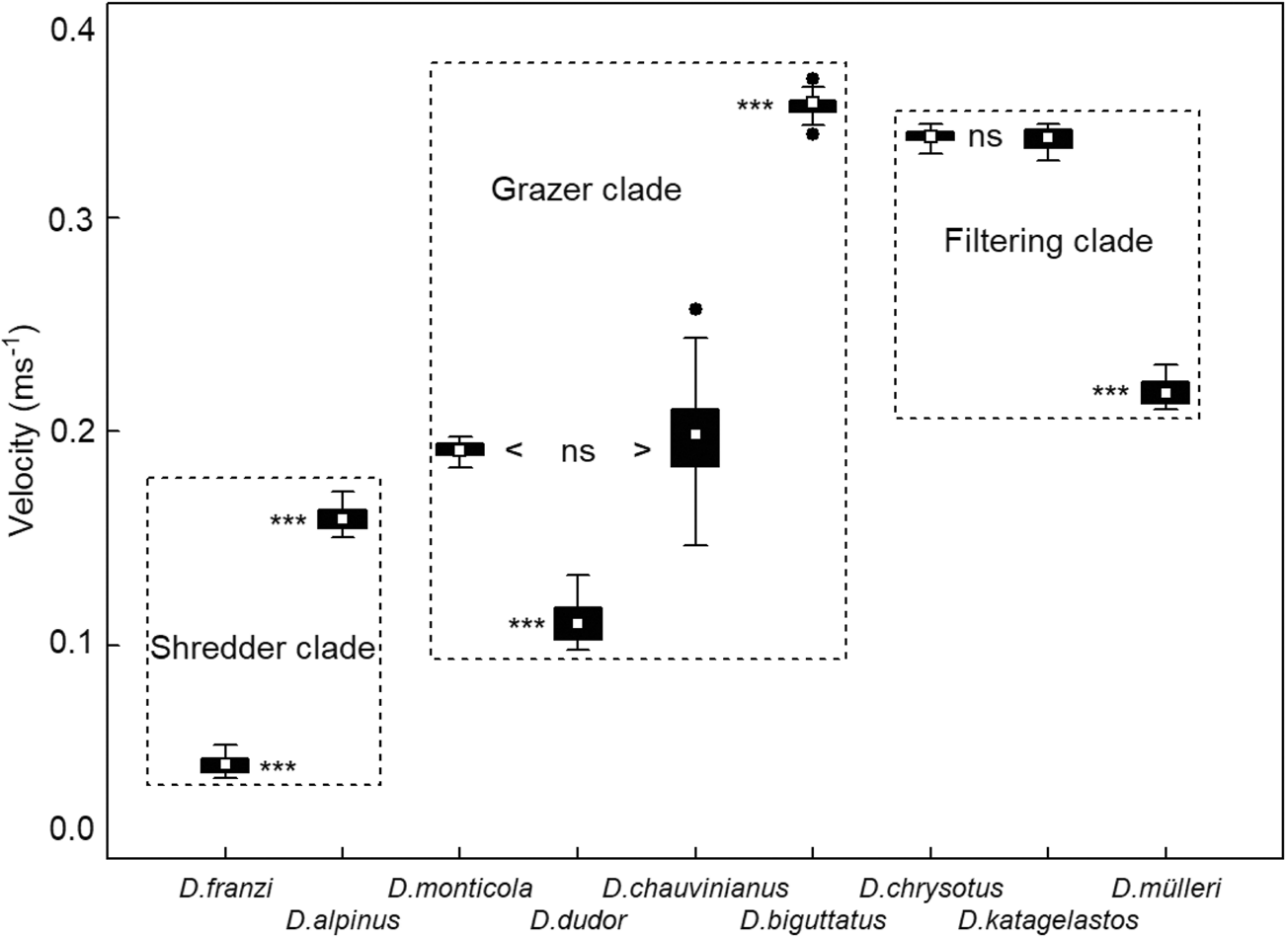
Flow velocity preferences (m s^−1^) of nine species of Drusinae, based on velocity measurements (intervals of 1 s over a period of 30 s) at front center of larvae (*N* = 68). White rectangles = means, black bars = 25/75% quartiles, whiskers = range without outliers, black dots = outliers. The differences between the three clades were highly significant (*p* = 0.000; Kruskal-Wallis ANOVA). Statistical differences between the species within a given clade (Mann-Whitney *U*-test) are indicated by the acronym ns (= not significant) or triple asterisks (very highly significant; *p* < 0.001)

The 68 stream velocity records (1 Hz samples) revealed series of smooth signals, combined with infrequent peaks during time periods of 1–3 s (Fig. 1). Two specimens of *Drusus franzi* Schmid, 1955 remained in a stagnant environment during the complete measuring run (Fig. 1, bottom). In detail, current speeds measured at the locations of the nine larval Drusinae ranged from 0 in *Drusus alpinus* Meyer-Dür, 1875, *D. franzi*, *D. chrysotus* (Rambur, 1842), *D. katagelastos* Vitecek, 2020, *D. mülleri* McLachlan, 1868, *D. dudor* Oláh, 2017 and *D. monticola* McLachlan, 1876 to 0.93 m s^−1^ recorded only once in *D. katagelastos*. The mean velocity (+ 95% confidence limits) for the three clades were 0.09 + 0.00 m s^−1^ for the shredder clade, 0.25 + 0.00 m s^−1^ for the grazer clade, and 0.31 + 0.01 m s^−1^ for the filtering carnivore clade. The differences between the three clades were very highly significant (*p* = 0.000; Kruskal-Wallis ANOVA). Within the shredder clade (Fig. 2), flow velocities at the sites of *D. alpinus* larvae were significantly higher than in *D. franzi* (Mann-Whitney *U*-test; *p* < 0.001). In the grazer clade, flow velocities were not significantly different (*p* > 0.05) in *D. monticola* and *D. chauvinianus* (Stein, 1874), but significantly (*p* < 0.001) lower in *D. dudor* and significantly higher in *D. biguttatus* (Pictet, 1834). Finally, in the filtering carnivore clade, flow velocities were not significantly different (*p* > 0.05) in *D. chrysotus* and *D. katagelastos*, but significantly (*p* < 0.001) lower in *D. mülleri* (Fig. 2).

**Fig. 3:**
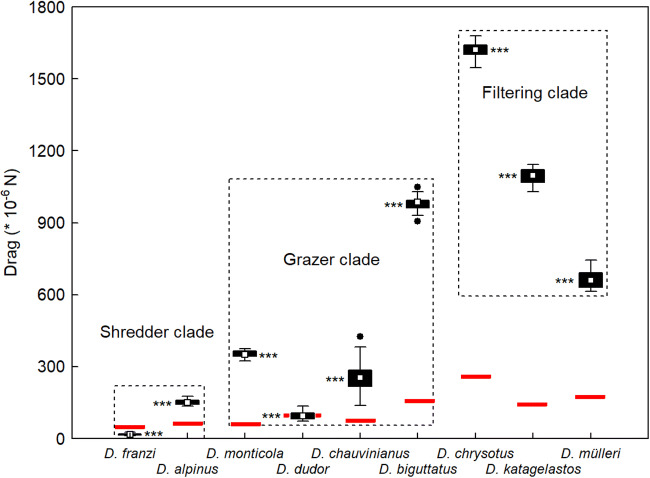
Box plots of hydraulic drag (10^−6^ N) exerted on final instar larvae of nine Drusinae species (= 68 specimens) with their longitudinal axis aligned with flow. Red bars indicate mean adhesive friction, based on species-specific submerged weight (10^−6^ N; Table 2). In *D. franzi* and some specimens of *D. dudor*, the observed drag was lower than or as high as adhesive friction, indicating that submerged weight by itself could fully stabilize the larvae. White rectangles = means, black bars = 25/75% quartiles, whiskers = range without outliers, black dots = outliers. The differences between the three clades were highly significant (*p* = 0.000; Kruskal-Wallis ANOVA). Statistical differences between the species within a given clade (Mann-Whitney *U*-test) were highly significant (*p* < 0.001) in all cases

Mean drag per data series ranged from 13*10^−6^ N in *D. franzi* to 1680*10^−6^ N in *D. chrysotus*; minima of 0 were observed at least once in all species except *D. chauvinianus*, whereas maxima of 8346 *10^−6^ N were recorded in *D. katagelastos* (Fig. 3). Hydraulic drag was only partly compensated by the submerged body mass of the larvae which ranged from 6.07 + 7.83 mg in *D. franzi* to 37.71 + 32.05 mg in *D. chrysotus*, equivalent to (41.07 + 53.03) *10^−6^ N and (255.24 + 216.87)*10^−6^ N, respectively (Table 2). Larvae had to actively withstand the remaining excess drag except in *D. franzi* larvae and in some larvae of *D. dudor* where adhesive friction was higher than drag (Fig. 3).

**Fig. 4:**
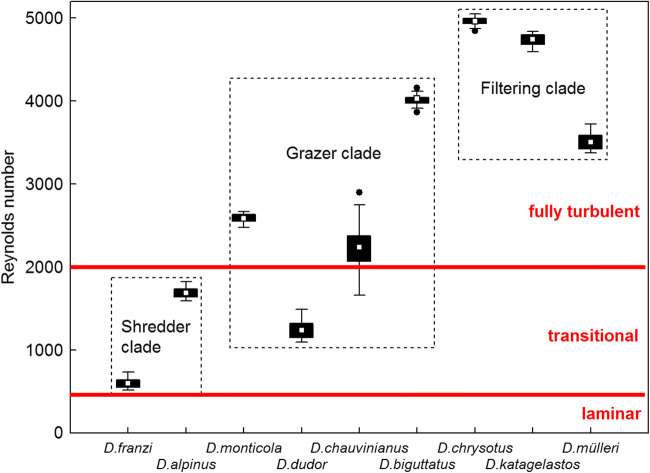
Box plots of organismic Reynolds numbers acting on fifth instar larvae of nine Drusinae species (= 68 specimens), heads directed upstream. Red bars indicate thresholds between laminar, transitional and fully turbulent regimes. The two species of the shredder clade and *D. dudor* were well in the transitional range (R = 500–2000), the rest of the grazer clade and the filtering clade species were in the fully turbulent range of R, with *D. chauvinianus* taking an intermediate position between transitional and fully turbulent. White rectangles = means, black bars = 25/75% quartiles, whiskers = range without outliers, black dots = outliers. The differences between the three clades were very highly significant (*p* = 0.000; Kruskal-Wallis ANOVA)

In the 68 larvae measured, Reynolds numbers (*R*) varied between 0 in *D. franzi* and *D. alpinus*, and 12,634 in *D. katagelastos*, with 7% of the total in the laminar range (*R* = 0–500), 30% in the transitional (*R* = 500–2000), and 61% in the fully turbulent stage (*R* > 2000). When broken down to clades, the percentages of laminar/transitional/fully turbulent were 31/50/19 in the shredder, 5/24/71 in the grazer, and 0/23/77 in the filtering carnivore clade, respectively. Mean *R* values at the species level are summarized in Fig. 4, with differences between the three clades being highly significant (Fig. 4).

**Fig. 5:**
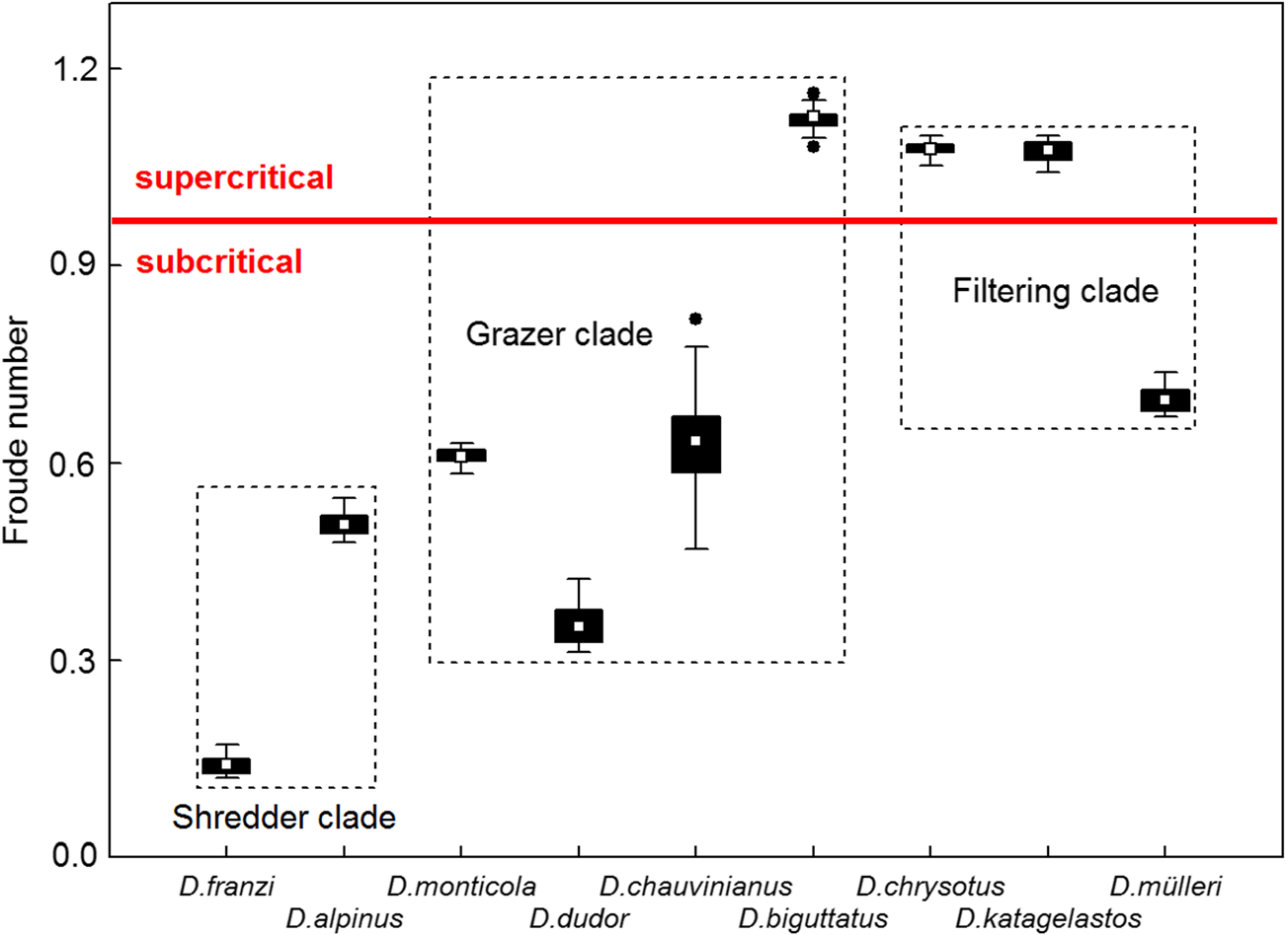
Box plots of Froude numbers at the locations of final instar larvae of nine Drusinae species (= 68 specimens). A red bar separates F values in the sub- and supercritical range. Only one species of the grazer clade and two species of the filtering clade were found to be in the supercritical range. White rectangles = means, black bars = 25/75% quartiles, whiskers = range without outliers, black dots = outliers. The differences between the three clades were very highly significant (*p* = 0.000; Kruskal-Wallis ANOVA)

Mean Froude numbers (+ 95% CL) ranged from 0 to 2.97 with mean *Fr* = 0.69 + 0.04. In the shredder clade, mean *Fr* were 0.33 + 0.05 and always in the subcritical range below 1. The latter was also true for *D. chauvinianus*, *D. dudor* and *D. monticola* of the grazer clade where *Fr* ranged from 0.31 to 0.82. In *D. biguttatus*, however, the mean Froude number was up to 1.12 + 0.01 (range = 1.08–1.16) and always in the supercritical range > 1 (Fig. 5). In filtering carnivores, *D. mülleri* was exposed to Froude numbers in the subcritical, the rest of the clade in the supercritical range (Fig. 5). The differences between the three clades were very highly significant (*p* < 0.001).

## Discussion

In this paper, the hydraulic parameters studied in detail are seen as n-dimensional subsets of the species-specific, multidimensional ecological niches of the nine Drusinae taxa. To illustrate this, stream velocity addresses fluid-mechanical effects on the larvae themselves, i.e. pressure and friction drag, but impacts as well, among others, respirational efficiency by replenishing oxygen-poor fluid layers in contact with gill epithelia (Vogel 1981). *R* reveals the dominance of pressure or friction drag in a given hydraulic field, thereby possibly triggering evolutionary adaptations for streamlining or body surface reduction (Statzner 1988; Statzner et al. 1988). Adhesive friction, on the other hand, elucidates the interplay of body volume and submerged weight, opening the door for morphological optimizations of ballast within a given body volume itself or by additional structures added to transportable Trichoptera cases (König and Waringer 2008). Finally, the ratio of inertial to gravitational forces is evaluated by *Fr*, defining, among others, whether surface waves can be used as a vector for transporting predator-released kairomons upstream, acting as warning substance for potential prey (Gordon et al. 1992; Peckarsky 1980). There may be even the case of gravity wave-induced downward lift forces on submerged bodies (Dawson 2014). Such examples illustrate the potential of hydraulic data for defining a significant fraction of ecological niche descriptors of aquatic biota (‘hydraulic niche’).

Stream velocities measured at the sites of the nine Drusinae species ranged from 0 to 0.93 m s^−1^; even at maximum current speeds, larvae remained firmly attached to the substrate, thereby avoiding the detrimental effects of erosion and subsequent drifting in the free water column (Waringer 1992). These data significantly exceed published upper thresholds of 0.28 m s^−1^ in fully-grown larvae of the caddisfly *Allogamus auricollis* (Pictet, 1834) (Waringer 1989a), but are lower than the maximum of 1.26 m s^−1^ reported for the Trichoptera genus *Silo* (König and Waringer 2008).

Hydrostatic lift due to Archimedes’ principle effectively reduced the submerged body mass (larva plus case) of the nine taxa investigated by 73–87% (Table 2). Therefore, on average, only 22% of hydraulic drag could be absorbed by weight alone in seven out of nine species; for the compensation of excess drag, those larvae had to rely on effectively clinging to the substrate, using their tarsal claws. Only in *D. franzi* and in some larvae of *D. dudor*, submerged weight alone fully compensated hydraulic drag (Fig. 3). In general, the 68 larvae investigated in detail were exposed to a mean drag of 581 *10^−6^ N. In other taxa, e.g. Goerid caddisflies, up to 40% of hydraulic drag could be absorbed by submerged body mass, thereby significantly increasing the range of larvae on the substrate without the danger of drift entry (Waringer 1989b; König and Waringer 2008).

Another force acting in a lotic environment is hydrodynamic lift due to pressure differences between sides, bottom and top of submerged bodies. Those forces significantly affect dorsoventrally flattened biota, e.g. ephemeropterans (Weissenberger et al. 1991), but were found to be negligible in taxa more or less cylindrical in shape (e.g., *Perla bipunctata* Pictet, 1833); the latter is also valid for Drusinae larvae in their cylindrical cases where hydraulic drag is much more important than hydrodynamic lift.

During larval growth, skin friction drag is dominant in early instars at low *R* values, but pressure drag successively becomes significant for older ones where *R* strongly increases (Vogel 1981). Consequently, Statzner et al. (1988) stressed the fact that a favourable surface-volume ratio is efficient for lowering drag at low *R* numbers, whereas streamlining can effectively reduce pressure drag at high *R*. The coverage of a wide range of Reynolds numbers induced by the high vagility of most aquatic biota at the sediment surface can consistently explain the high number of morphologies combining (hemi-)spherical and streamlined shapes.

In the nine *Drusus* species investigated, mean *Fr* was 0.69 + 0.04 (subcritical). At the species level, however, there were large differences: the shredder clade and the majority of the grazer clade were always exposed to *Fr* in the subcritical range below 1, whereas the locations of *D. biguttatus* and the majority of the filtering clade were well in the supercritical range. In fact, *Fr* numbers >1 are favourable for filter-feeding Drusinae larvae inhabiting shallow microhabitats exposed to high stream velocities, whereas Froude numbers <1 are preferred by taxa constructing filtering nets, e.g., Hydropsychidae (Wetmore et al. 1990).

**Fig. 6:**
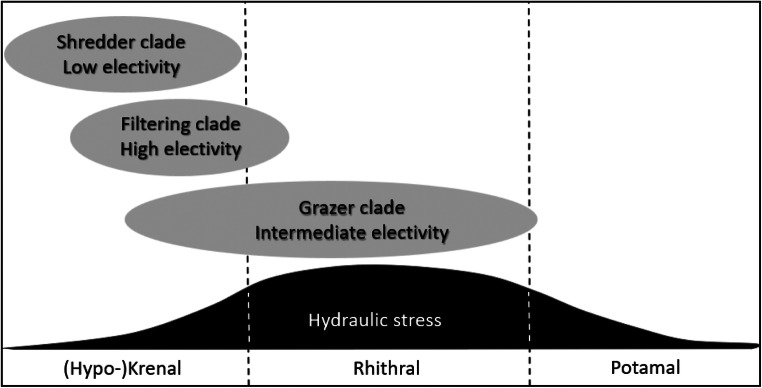
Conceptual model of longitudinal zonation patterns and hydraulic stress exposition of the three Drusinae clades, in accordance with data given in Figs. 2–5. Shredders prefer hydrodynamic low stress patches within their predominantly low-stress (hypo-)krenal habitats, in accordance with their food items concentrated near the banks (accumulations of coarse particulate organic matter, roots and riparian grass), resulting in a low electivity of microhabitats. Grazers are restricted to hydrodynamic medium to high stress patches in accordance with autotrophic biofilms and epilithic algae concentrated in off-bank sections; as such conditions are predominant in their rhithral habitats, this results in an intermediate electivity of microhabitats. Species of the filtering clade, however, are most abundant in low-stress (hypo-)krenal habitats, but they rely on high stress patches because high flow velocity is required to efficiently operate their filtering apparatus; this results in a high electivity of the few high-velocity patches available. Predicted hydrodynamic stress along the stream continuum according to Statzner and Higler 1986; longitudinal zonation patterns of Drusinae clades are based on species-specific valency point distribution data given by Graf et al. (2008)

Valency point distribution scores for longitudinal zonation patterns within the stream continuum included in Table 1 (Graf et al. 2008; Vitecek et al. 2020)) were established by caddisfly experts and can be seen as a proxy of hydraulic stress at the microhabitats of the nine *Drusus* species. Along a standard pristine running water course, predicted hydrodynamic stress at the source and the spring brooks is low (section A), becomes high at the adjacent steep-gradient headwater reach (section B), and levels off again further downstream to the mouth (section C; Statzner and Higler 1986). In European highland streams, section A represents the Krenal and Hypokrenal, section B the Rhithral, and section C the Potamal (Illies 1961; Illies and Botoșaneanu 1963; Statzner and Higler 1986) (Fig. 6). For the nine Drusinae species studied in detail, no valency points were attributed to the Potamal at all. Furthermore, for the two species included in the shredder clade, all ten valency points available were included within the (Hypo-)krenal section but none in the Rhithral category, suggesting a predominance of low hydraulic stress in their hydraulic niches. This is in accordance with our field measurements (Figs. 2–5). Low flow velocities are prerequisites of particulate organic matter accumulation, and near-stagnant niches are also concentrated near the banks where riparian grass vegetation acts as another important food item for shredders such as *D. franzi* and *D. alpinus*. The valency point distribution for the grazer clade is rather diverse, reflecting a broad spectrum of longitudinal zonation patterns from purely spring and springbrook species (*D. dudor*) to rhithral species such as *D. chauvinianus* and *D. biguttatus*, with *D. monticola* taking an intermediate position (Table 1). Data reflect this by yielding the widest range of hydraulic stress descriptors of all three clades (Figs. 2–5). As expected from valency point distribution (Table 1), *D. dudor* is close to the shredder clade whereas the other three species are close to the filtering carnivore clade. The hydraulic niches chosen by the grazer clade reflect the fact that autotrophic biofilms and epilithic algae are concentrated at lotic patches off-banks in larger streams (König and Waringer 2008), but extend also to more lenitic patches in narrow springbrooks. The high share of Krenal and Hypokrenal valency points in the filtering carnivore clade (Table 1) suggests a low proportion of high velocity patches in those springs and springbrooks. Nevertheless, filter feeders were exposed to the highest hydraulic stress of all three clades, because high flow velocities are required to efficiently operate their filtering apparatus. This also mirrors the high electivity of filter feeders for the few lotic patches available in spings and springbrooks (Fig. 6), where they accumulate in high densities for optimizing their foraging strategies (Graf et al. 2005; Vitecek et al. 2015).
